# Severe Bilateral Hyperkeratosis of the Nipples and Areolae: A Case Report and Literature Review

**DOI:** 10.3389/fmed.2022.781693

**Published:** 2022-02-23

**Authors:** Jiaying Wei, Qingshu Li, He Wu, Xuedong Yin, Guosheng Ren

**Affiliations:** ^1^Department of Endocrine and Breast Surgery, The First Affiliated Hospital of Chongqing Medical University, Chongqing, China; ^2^Department of Pathology, Chongqing Medical University, Chongqing, China; ^3^Department of Thyroid and Breast Surgery, Xi'an No. 3 Hospital, The Affiliated Hospital of Northwest University, Xi'an, China

**Keywords:** hyperkeratosis of the nipples and areolae, RNA-sequencing, Dermatology, hyperkeratosis, genomic alteration

## Abstract

**Background:**

Hyperkeratosis of the nipple and areola (HNA) is a rare skin disease with unknown etiology. Some patients are misdiagnosed or never diagnosed, especially during the early stage of this disease. In addition, the mechanism involved in the development of HNA is still unknown, and genomic alterations have not been reported anywhere.

**Case Information:**

A 26-year-old female suffered gradual bilateral areola thickening and enlargement, with accompanying intense itching, and was diagnosed with HNA at the First Affiliated Hospital of Chongqing Medical University. No obvious abnormalities were found in laboratory test examinations such as hormone testing for estrogen, progesterone, or prolactin. Typical papillomatous skin with orthokeratotic hyperkeratosis and numerous infiltrating lymphocytes was detected through a histopathological examination. The results from RNA-sequencing showed that the molecular expression between HNA and a normal nipple and areola (NNA) was obviously different. No significant difference was found in the bilateral lesions. In addition, immune-related cell signaling pathways were overactivated in HNA compared to the control HNA.

**Conclusion:**

The typical symptoms, clinical features, and histopathological alterations presented in this case lead to a profound understanding of HNA, which can avoid the misdiagnosis and missed diagnosis of this disease at an early stage. The dysfunction of the local immune system, which was demonstrated by pathological examination and genomic analysis, suggests that anti-autoimmune therapy, such as steroid medication, may be an effective treatment for HNA at an early stage.

## Introduction

Hyperkeratosis of the nipple and areola (HNA) is a rare skin disease of the areola and/or nipples and was first introduced by Tauber in 1923 Oberste-Lehn (1, 2). HNA is not a malignant disease, but symptoms frequently occur, such as severe pruritus and purulent discharge. Moreover, the appearance of the breast can be disfigured by HNA which affects self-confidence in these patients. To date, only ~121 cases of HNA have been reported worldwide (3), ~80% of which occurred in women aged 20–30 (4–6) (Table 1). The etiology of HNA remains unclear. Only one retrospective study proposed that abnormal serum estrogen levels are associated with HNA (7). This conclusion needs to be demonstrated in more studies.

**Table 1:** Characteristics of the patients included in the study (*n* = 121) by age^a^.

**Baseline characteristic**	**Age groups (*****n*** **=** **8,594)**							
	** <10 years**	**10–20 years**	**20–30 years**	**30–40 years**	**40–50 years**	**50–60 years**	**≥60 years**	
	**(*n* = 1)**	**(*n*=20)**	**(*n* = 460)**	**(*n* = 32)**	**(*n* = 6)**	**(*n* = 5)**	**(*n* = 11)**	***P* value ^b^**
**Sex**
Female (*n* = 99)	1 (1.0%)	19 (19.2%)	42 (42.4%)	29 (29.3%)	4 (4.0%)	3 (3.0%)	1 (1.0%)	**<0.001**
Male (*n* = 22)	0 (0.0%)	1 (4.5%)	4 (18.2%)	3 (13.6%)	2 (9.1%)	2 (9.1%)	10 (45.5%)	
**Diseased side**
Unilateral (*n* =35)	0 (0.0%)	7 (20.0%)	10 (28.6%)	8 (22.9%)	4 (11.4%)	3 (8.6%)	3 (8.6%)	**0.192**
Bilateral (*n* =86)	1 (1.2%)	13 (15.1%)	36 (41.9%)	24 (27.9%)	2 (2.3%)	2 (2.3%)	8 (9.3%)	
**Complication**
Without (*n* =104)	1 (1.0%)	20 (19.2%)	41 (39.4%)	29 (27.9%)	5 (4.8%)	3 (2.9%)	5 (4.8%)	**0.001**
With (*n* =17)	0 (0.0%)	0 (0.0%)	5 (29.4%)	3 (17.6%)	1 (5.9%)	2 (11.8%)	6 (35.3%)	
**Levy-Tranckel**
**classification ^c^**
I (*n* =29)	0 (0.0%)	8 (27.6%)	11 (37.9%)	7 (24.1%)	0 (0.0%)	1 (3.4%)	2 (6.9%)	**0.162**
III (*n* =92)	1 (1.1%)	12 (13.0%)	35 (38.0%)	25 (27.2%)	6 (6.5%)	4 (4.3%)	9 (9.8%)	
**Mehanna**
**classification ^d^**
I (*n* =7)	0 (0.0%)	0 (0.0%)	3 (42.9%)	1 (14.3%)	0 (0.0%)	0 (0.0%)	3 (42.9%)	**0.046**
II (*n* =74)	0 (0.0%)	12 (16.2%)	23 (31.1%)	25 (33.8%)	4 (5.4%)	3 (4.1%)	7 (9.5%)	
III (*n* =40)	1 (2.5%)	8 (20.0%)	20 (50.0%)	6 (15.0%)	2 (5.0%)	2 (5.0%)	1 (2.5%)	

a *Values are given as number (percentage)*.

b *Some P values were compared using χ^2^ tests*.

c *Cut-off values of Levy-Tranckel classification were defined as follows: (1) epidermal nevus, with unilateral involvement of a single breast, usually in a Blaschko linear pattern (observed equally in both genders); (2) hyperkeratosis of the nipple and areola associated with ichthyosis, with bilateral involvement of the breasts (observed equally in both genders); and (3) a bilateral type, generally affecting both nipples and areolae and observed mainly in females in the second or third decade of life*.

d *Cut-off values of Mehanna classification were defined as follows: (1) primary HNA occurring coincidentally with other dermatoses such as acanthosis nigricans, ichthyosis, or Darier's disease; (2) secondary HNA caused by hormonal changes, malignancies, or lymphoma; and (3) idiopathic HNA without any obvious cause*.

In 1938, Levy-Frenckel divided HNA into 3 types as follows. Type I HNA represents the extension of the verrucous nevus, which is usually unilateral. Type II HNA, associated with disseminated dermatoses such as ichthyosis, lymphoma, AN, and Darier's disease, and type III idiopathic HNA are also known as nevoid hyperkeratosis. The first subtype is epidermal nevus with the unilateral involvement of a single breast, usually in a Blaschko linear pattern (observed equally in both genders). The second subtype is HNA, which is considered a complication of ichthyosis, involving bilateral breasts and is observed equally in both sexes. The third subtype is a bilateral type, generally affecting both nipples and areolae and observed mainly in females in the second or third decade of life (8). The latest classification proposed by Mehanna divides HNA into three types: (1) primary HNA that occurs simultaneously with other dermatoses such as acanthosis nigricans, ichthyosis, or Darier's disease; (2) secondary HNA caused by hormonal changes, malignancies, or lymphoma; and (3) idiopathic HNA with no obvious cause (9). Here, a patient with bilateral HNA of typical secondary type in Mehanna classification and the third type nevus-like in Levy-Tranckel classification and its clinical characteristics, including histological detection will be introduced. The genomic analysis will be performed to investigate the mechanism involved in the development of HNA. In addition, the therapeutic strategies for the treatment of HNA will be discussed. This case report follows the CARE Guidelines (10).

## Patient Information

A 26-year-old female was admitted to the hospital on August 17, 2020. The patient first noticed the changes in the nipple-areola complex before puberty onset. Eight years ago, the patient complained of gradual bilateral areola thickening and enlargement, with accompanying intense itching. Biopsies of the region were performed at the Dermatology Department of the Eighth Affiliated Hospital of Sun Yat-Sen University. A histopathological diagnosis of the squamous papilloma of the areolae was reported. Symptoms worsened over time, including scabbing of the nipple-areola area, severe pruritus, and non-purulent discharge. These symptoms had a negative impact on the patient's physiological and psychological wellbeing. The patient was unmarried and not pregnant, with a regular menstrual cycle. The patient had no personal or family history of atopic dermatitis or ichthyosis vulgaris. Her father died of lung cancer, and her mother was diagnosed with lymphoma. For a further biopsy, this patient was admitted to the First Affiliated Hospital of Chongqing Medical University. Pathological examination revealed bilateral hyperkeratosis of the nipples.

## Clinical Findings

**Figure 1:**
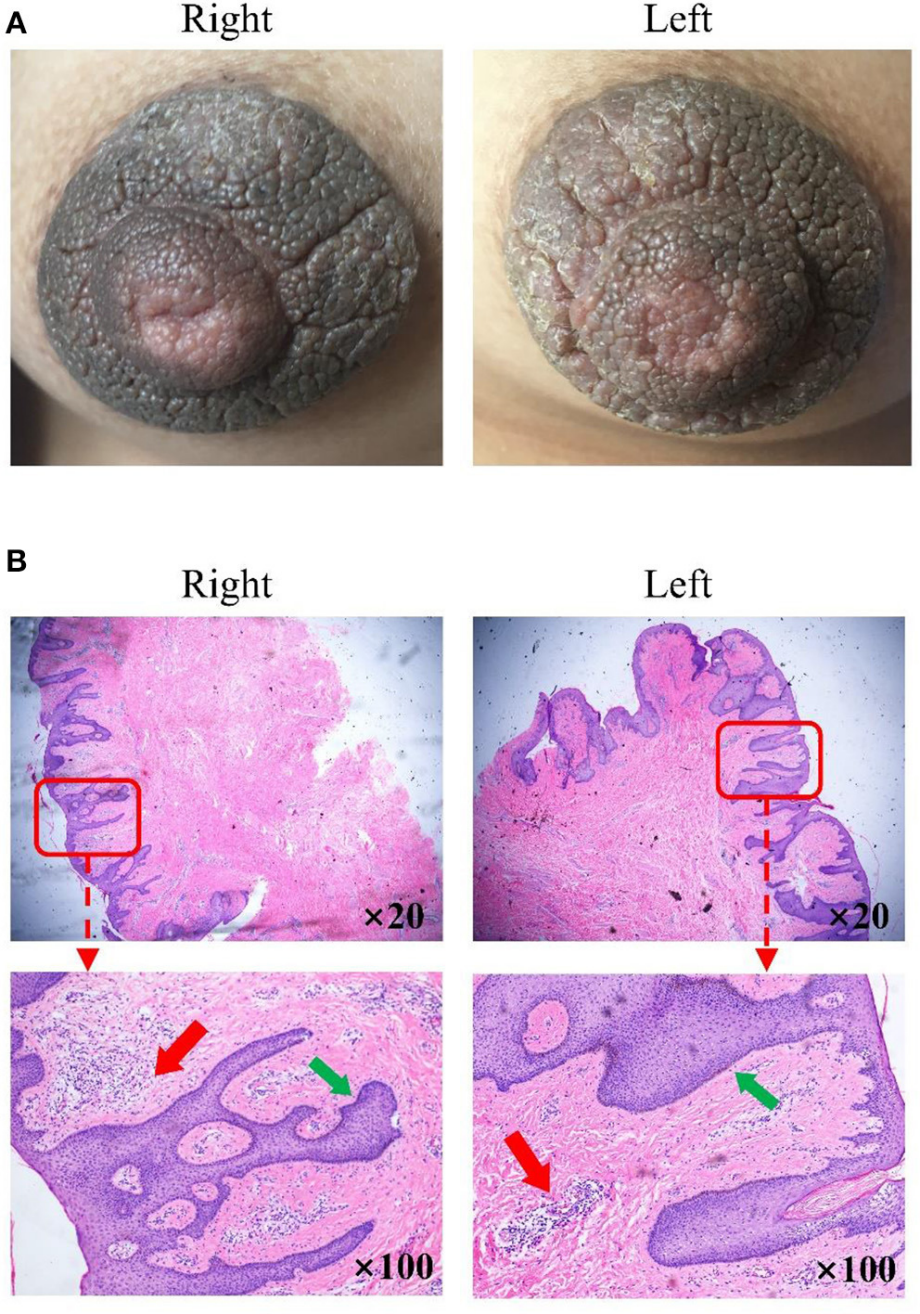
Clinical images and histological detection of hyperkeratosis of the nipple and areola (HNA). **(A)** Nevoid hyperkeratosis-like lesion of the nipple and areola in the right and left mammary areas. **(B)** Stained sections were observed under a fluorescence microscope magnification in the right and left mammary areas. Hematoxylin-eosin stain; original magnification, ×20 and ×100. The thin red arrow indicates immune cell infiltration in a significant number of samples. The thin green arrow indicates epidermal hyperplasia was also evident.

Physical examination revealed bilateral pigmentation, thickened nipples and areolas, and deepened skin texture (Figure 1A). The nipple-areola complex was thickened with hyperkeratosis and more protuberant. No tenderness was reported upon palpation, and no bleeding or discharge was observed. The lesions of the areolae around the nipples were ~2 cm in diameter with clear boundaries.

The patient's laboratory examination, including routine blood, urine tests, and hormone tests, was normal. Abdominal, thyroid, and breast B-ultrasound revealed no obvious abnormalities.

The examination of the stained section under a light microscope (Figure 1B) revealed papillomatous skin with orthokeratotic hyperkeratosis and follicular plugging, alongside acanthosis. Light microscopic examination of the skin lesions revealed epidermal hyperkeratosis, follicle keratinization, and spinous layer hypertrophy. Elongated rete pegs suggested the presence of papillomatous hyperplasia. An increase in melanin in the basal layer was identified alongside tortuous capillaries in the dermal papilla and superficial perivascular mixed lymphoeosinophilic infiltrate.

**Figure 2:**
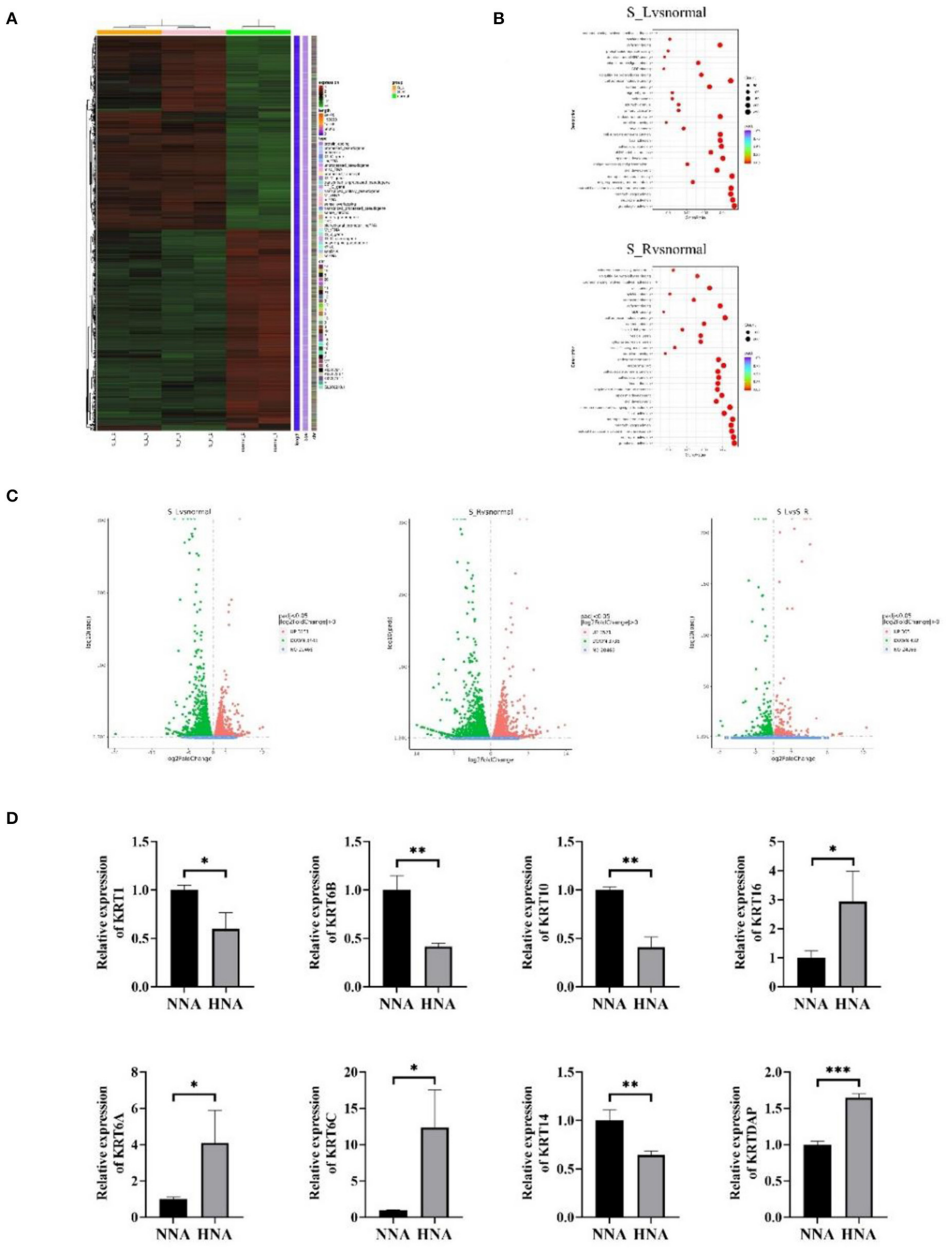
Genomic expression detected by RNA-sequencing performed between the HNA and normal nipple and areola (NNA). **(A)** The heat map of the genomic expression cluster. Group S-L means the left mammary area. Group S-R means the right mammary area. Group normal means the mammary area from the normal patient. **(B)** The enrichment analysis aimed at detecting the variation of the cell signaling pathways and biological functions among different groups in the right and left mammary areas. **(C)** The gene expression of the lesions from both sides. The difference in gene expression between the lesions from both sides was not obvious, while the difference in gene expression between the lesions and healthy samples was obvious. **(D)** Keratins and their associated proteins are dysregulated in HNA compared to NNA. ^*^P < 0.05, ^**^P < 0.01, ^***^P < 0.001.

Genomic expression was detected by the RNA-sequencing performed between the lesions from HNA and the control lesions from normal nipple and areola (NNA) (Figure 2). The methodology used for genome expression was Paraffin embedding (PFFE) and second-generation sequencing. The heatmap of the genomic expression cluster showed that the transcriptional messenger RNA (mRNA) expression in HNA was significantly different from that in NNA (Figure 2A). However, the difference in gene expression between the lesions from both sides was not obvious (Figure 2C). The enrichment analysis which aims at detecting the variation of cell signaling pathways and biological functions showed that immune-related functions were significantly affected by HNA disease compared to the control functions (Figure 2B). In addition, keratins and their associated proteins were also dysregulated in HNA compared to NNA (Figure 2D).

## Diagnostic Assessment and Diagnosis

In summary, the patient was diagnosed with bilateral hyperkeratosis of the nipples and areola.

The major characteristics of HNA are pigmentation and thickening of the nipples and areolae with deepening skin texture and the absence of tenderness on palpation, bleeding, or discharge. The differential diagnoses of HNA include Pagets' disease of the nipple, epidermal nevus, acanthosis nigricans, chronic dry papular eczema, localized breast Darier's disease, and condyloma acuminatum of the nipple (5). To differentiate HNA from other diseases, pathological examination of the tissue is necessary. The gene sequencing results are consistent with the pathological findings of this patient.

## Therapeutic Interventions and Follow-Up and Outcomes

The patient was prescribed Tretinoin 0.05% cream for topical application, but no improvement was noted after 7 months of treatment. No other treatments were attempted after the ineffectivity of the Tretinoin 0.05% cream.

## Patient Perspective

“I did not want to delay future lactation because of treatment such as surgery or hormonal drugs, and I would like to try Ellman's radiofrequency current after regular birth and lactation, but also worried about the possibility of disease deterioration caused by hormonal changes in pregnancy and perinatal period”.

## Discussion

Hyperkeratosis of the nipple and areola (HNA) is a rare benign skin disease of the areola or nipples. However, most patients showed no typical symptoms of HNA at an early stage. The pathogenesis of unilateral and bilateral disease may represent distinct genetic causes: unilateral pathogenesis suggests localized mutation, while bilateral disease suggests germline mutation. Here, we present an HNA case with severe bilateral skin abnormalities. There was no history of pain, ulceration, bleeding, or any symptoms of the nipple and areola since adolescence, except hyperkeratosis. After the hot and humid summer weather and the friction between underwear, symptoms worsened to include severe pruritus and purulent discharge. This is a typical secondary type according to the Mehanna classification. The patient did not present with other skin diseases such as ichthyosis or epidermal nevus. According to the Levy-Tranckel classification, this case belongs to the third type nevus-like type, which is the most common type and occurs in women aged from 20 to 30. This type presents bilateral lesions. Due to the obvious family history of cancer, she may carry some new germline mutations.

Hyperkeratosis of the nipple and areola (HNA) typically develops in late adolescence or early adulthood (11). The etiology of this disease is still unknown (12). HNA has previously been suggested as an adverse effect of BRAF inhibitors during the treatment of metastatic malignant melanoma (13). However, most cases of HNA developed from puberty, suggesting a possible link to hormonal stimulation. The hormone level was normal in this case; however, the hormone-related factors could be dynamically changed. Thus, it is suggested that hormone factors should be monitored at the very early stage of this disease. Previous studies have shown that some genes, including the keratin family, are dysregulated in HNA (14). This disease is caused by keratin hyperplasia closely related to abnormalities in keratin expression. We wanted to see which specific subtype of keratin expression has changed (15). To explore the mechanism that may be involved in the development of HNA, RNA sequencing was carried out to detect alterations in genomic expression in HNA. Biopsy specimens from the NNA were used as the control. Whole-genome analysis indicated that the gene expression in HNA was tremendously different from that in NNA. Interestingly, the genomic expression of the samples from the right lesion was not significantly different from that of the left lesion. This result may support the concept that inherited or acquired genetic mutation (in the development of embryogenesis) could lead to simultaneous bilateral HNA. However, no evidence had been reported anywhere. Pathological examination indicated that the immune response was upregulated in HNA because many infiltrating immune cells were detected under the epidermis. This histological alteration was further proven by genomic differentiation analysis, which showed that immune-related biological functions such as neutrophil and granulocyte activation were upregulated in HNA compared to NNA. It is speculated that HNA may be an autoimmune disease, and steroid medication could be one of the therapeutic strategies for HNA. Many autoimmune diseases typically improve with hormonal therapy (16, 17). In addition, the expression of the keratin family was confirmed to be dysregulated, increases in KRT6A, KRT6C, KRT16, and KRTDAP and decreases in KRT1, KRT6B, KRT10, and KRT14 (Figure 2D).

Due to the lack of knowledge about HNA, it is difficult to make a correct diagnosis of this disease at an early stage. The major characteristics of HNA are pigmentation and thickening of the nipples and areolae with deepening skin texture (5) and the absence of tenderness on palpation, bleeding, or discharge. To differentiate HNA from other diseases, pathological examination of the tissue is necessary (18). Current treatments indicated in previous studies include the application of medications such as glucocorticoid ointment, physiotherapy, and surgical operations such as skin lesion curettage (5, 19, 20).

## Conclusion

The typical symptoms, clinical features, and histopathological alterations presented in this case lead to a profound understanding of HNA, which can avoid the misdiagnosis and missed diagnosis of this disease at an early stage. In this case, the pathological analysis showed hyperpigmentation in the germinal layer at the base of the epidermis. Epidermal squamous epithelial hyperplasia was limited to the epidermis. A large number of lymphocytes proliferate and infiltrate under the epidermis, suggesting a potential sensitivity to hormonal drugs, because the inflammatory cells present in the inflammatory infiltrate are mixed lymphoeosinophilic. The results from the genomic analysis suggest the possibility of anti-autoimmune therapy for HNA. In addition, it was noted that the skin at the puncture site returned to normal after the biopsy, and no recurrence was found. This observation supports one opinion that surgery should be an effective treatment for severe HNA.

## Data Availability Statement

The original contributions presented in the study are included in the article/supplementary materials, further inquiries can be directed to the corresponding author/s.

## Ethics Statement

The study was approved and supervised by the Ethics Committee of the First Affiliated Hospital of Chongqing Medical University. The patients/participants provided their written informed consent to participate in this study. Written informed consent was obtained from the individual(s) for the publication of any potentially identifiable images or data included in this article.

## Author Contributions

All authors listed have made a substantial, direct, and intellectual contribution to the work and approved it for publication.

## Conflict of Interest

The authors declare that the research was conducted in the absence of any commercial or financial relationships that could be construed as a potential conflict of interest.

## Publisher's Note

All claims expressed in this article are solely those of the authors and do not necessarily represent those of their affiliated organizations, or those of the publisher, the editors and the reviewers. Any product that may be evaluated in this article, or claim that may be made by its manufacturer, is not guaranteed or endorsed by the publisher.

## References

[1] Oberste-Lehn H. Hyperkeratosis in the region of the nipple and areola. Z Haut Geschlechtskr. (1950) 8:388–93. 15431803

[2] Kartal Durmazlar SP Eskioglu F Bodur Z. Hyperkeratosis of the nipple and areola: 2 years of remission with low-dose acitretin and topical calcipotriol therapy. J Dermatolog Treat. (2008) 19:337–40. 10.1080/09546630802051645 18608736

[3] Riley CA Badri T Hafsi W. Hyperkeratosis of the Nipple Areola. Treasure Island: StatPearls (2020). Available online at: https://www.ncbi.nlm.nih.gov/books/NBK459144/ (accessed February 10, 2022).

[4] Fenniche S Badri T. Images in clinical medicine. Nevoid hyperkeratosis of the nipple and areola. New Engl J Medicine. (2010) 362:1618. 10.1056/NEJMicm0909467 20427810

[5] Aytekin S Tarlan N Alp S Uzunlar AK. Naevoid hyperkeratosis of the nipple and areola. J Eur Acad Dermatol Venereol. (2003) 17:232–3. 10.1046/j.1468-3083.2003.00577_5.x 12705764

[6] Baykal C Büyükbabani N Kavak A Alper M. Nevoid hyperkeratosis of the nipple and areola: a distinct entity. J Am Acad Dermatol. (2002) 46:414–8. 10.1067/mjd.2002.119646 11862178

[7] Kuhlman DS Hodge SJ Owen LG. Hyperkeratosis of the nipple and areola. J Am Acad Dermatol. (1985) 13:596–8. 10.1016/S0190-9622(85)70203-4 4078050

[8] Ghanadan A Balighi K Khezri S Kamyabhesari K. Nevoid hyperkeratosis of the nipple and/or areola: treatment with topical steroid. Indian J Dermatol. (2013) 58:408. 10.4103/0019-5154.117347 24082214 PMC3778809

[9] Mehanna A Malak JA Kibbi AG. Hyperkeratosis of the nipple and areola: report of 3 cases. Arch Dermatol. (2001) 137:1327–8. 10.1001/archderm.137.10.1327 11594857

[10] Riley DS Barber MS Kienle GS Aronson J von Schoen-Angerer T Tugwell P. CARE explanation and elaborations: reporting guidelines for case reports. J Clin Epidemiol. (2017) 89:218–35. 10.1016/j.jclinepi.2017.04.026 28529185

[11] Soriano LF Piansay-Soriano ME. Naevoid hyperkeratosis of the nipple and areola: an extensive form in two adolescent Filipino females. Clin Exp Dermatol. (2015) 40:23–6. 10.1111/ced.12461 25251504

[12] Kavak A Parlak AH Aydogan I. Hyperkeratosis of the nipple and areola in a patient with chronic mucocutaneous candidiasis. J Dermatol. (2006) 33:510–1. 10.1111/j.1346-8138.2006.00122.x 16848830

[13] Carr ES Brown SC Fiala KH. Painful nipple hyperkeratosis secondary to vemurafenib. Dermatol Therapy. (2017) 30:12477. 10.1111/dth.12477 28211633

[14] Sander D Dietmaier W Wobser M Haferkamp S. Hyperkeratosis of the nipple and areola: a histopathologic pitfall. J Dtsch Dermatol Ges. (2018) 16:1368–70. 10.1111/ddg.13677 30295995

[15] Yang L Fan X Cui T Dang E Wang G. Nrf2 promotes keratinocyte proliferation in psoriasis through up-regulation of keratin 6, keratin 16, and keratin 17. J Invest Dermatol. (2017) 137:2168–76. 10.1016/j.jid.2017.05.015 28576737

[16] Guevara-Gutiérrez E Tarango-Martínez VM Sandoval-Tress C Hernández-Torres M. Hiperqueratosis nevoide del pezón y la areola unilateral tratada con calcitriol tópico [Unilateral nevoid hyperkeratosis of the nipple and areola treated with topical calcitriol]. Actas Dermosifiliogr. (2008) 99:500–1. 10.1016/S0001-7310(08)74733-1 18558069

[17] Mold DE Jegasothy BV. Estrogen-induced hyperkeratosis of the nipple. Cutis. (1980) 26:95–6. 7389406

[18] Sengül N Parlak AH Oruk S Boran C. Nevoid hyperkeratosis of the nipple and areola: a diagnosis of exclusion. Breast J. (2006) 12:383–4. 10.1111/j.1075-122X.2006.00284.x 16848855

[19] Bayramgürler D Bilen N Apaydin R Erçin C. Nevoid hyperkeratosis of the nipple and areola: treatment of two patients with topical calcipotriol. J Am Acad Dermatol. (2002) 46:131–3. 10.1067/mjd.2002.117848 11756960

[20] Ozyazgan I Kontaş O Ferahbaş A. Treatment of nevoid hyperkeratosis of the nipple and areola using a radiofrequency surgical unit. Dermatol Surg. (2005) 31:703–5. 10.1097/00042728-200506000-00017 15996425

