# Supplementary material for: Severe Bilateral Hyperkeratosis of the Nipples and Areolae: A Case Report and Literature Review
Source: Front Med (Lausanne). 2022 Feb 23;9:781693. doi: 10.3389/fmed.2022.781693 (PMC8905514; doi:10.3389/fmed.2022.781693)
Supplement: Supplementary file 1 [file Data_Sheet_1.docx]

Supplemental material of *Severe* *bilateral hyperkeratosis of the nipples and areolae*


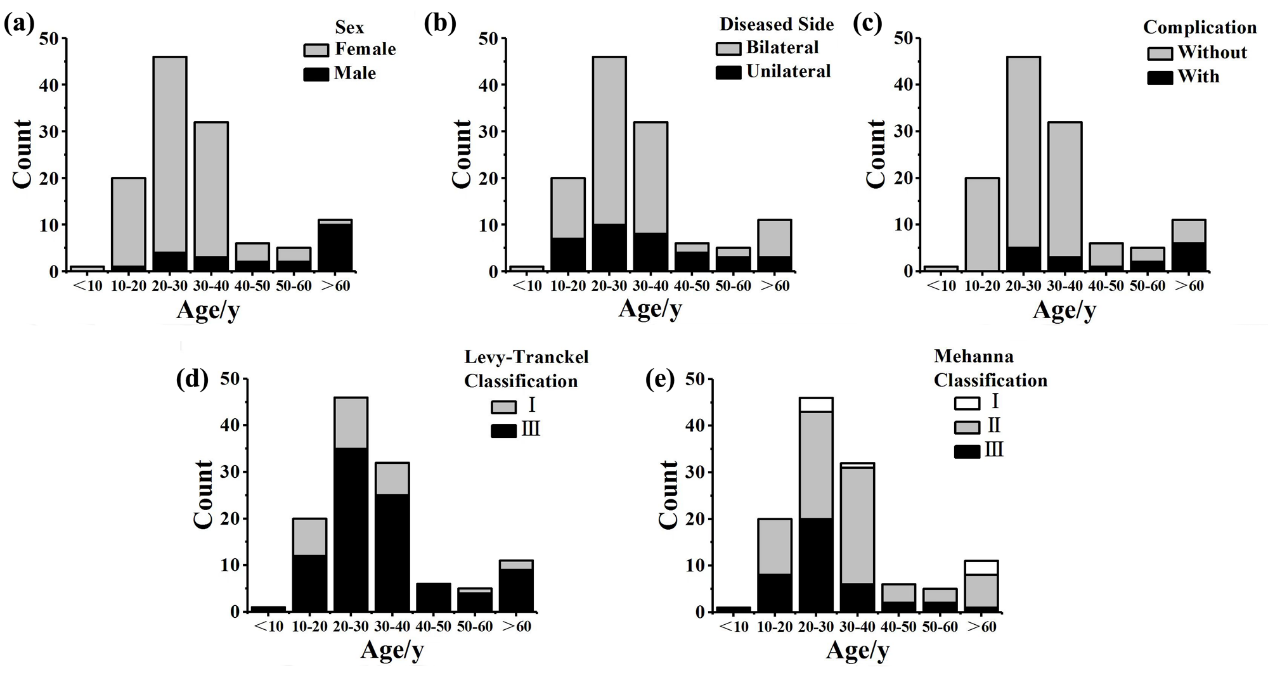


Supplemental Fig. 1. Sex, Diseased side, Complication, Levy-Tranckel classification and Mehanna classification in different age groups. (121 patients had developed the HNA since 1936)


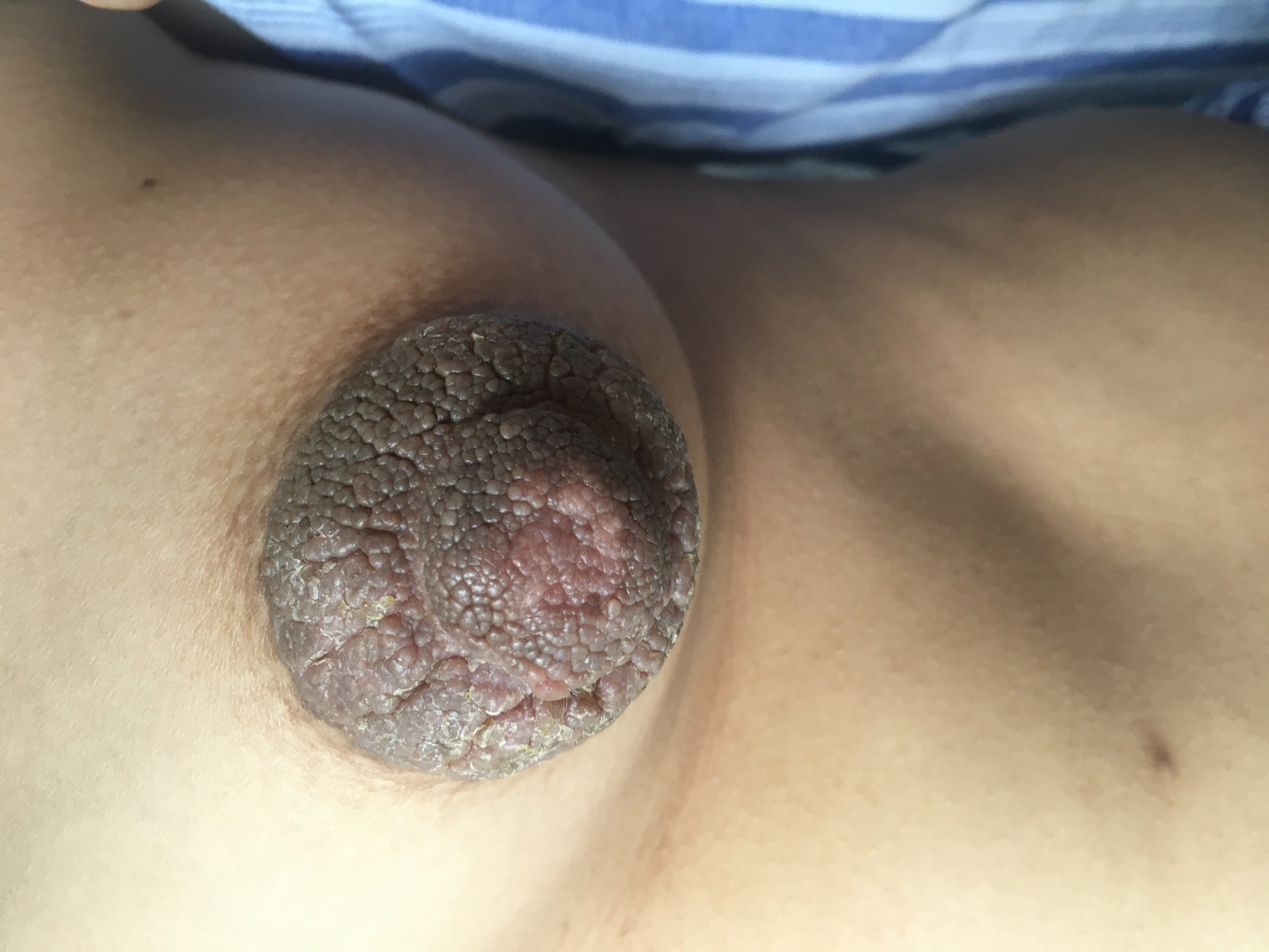


Supplemental Fig. 2. Nevoid hyperkeratosis-like lesion of the nipple and areola in the left mammary area.


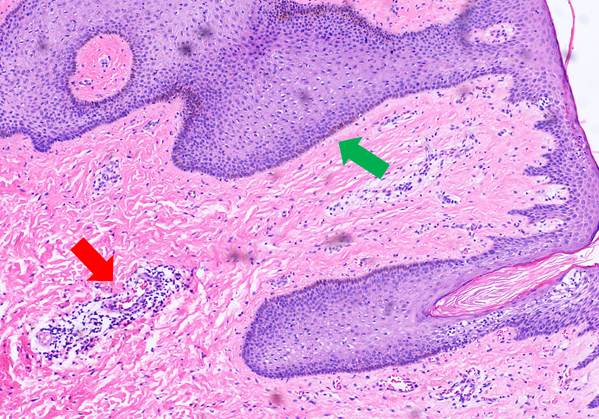


Supplemental Fig. 3. Stained sections were observed under a fluorescence microscope magnification in the left mammary area. Hematoxylin-eosin stain; original magnification, × 100. The thin red arrow in indicates immune cell infiltration in a significant number of samples. The thin green arrow in indicates epidermal hyperplasia was also evident.


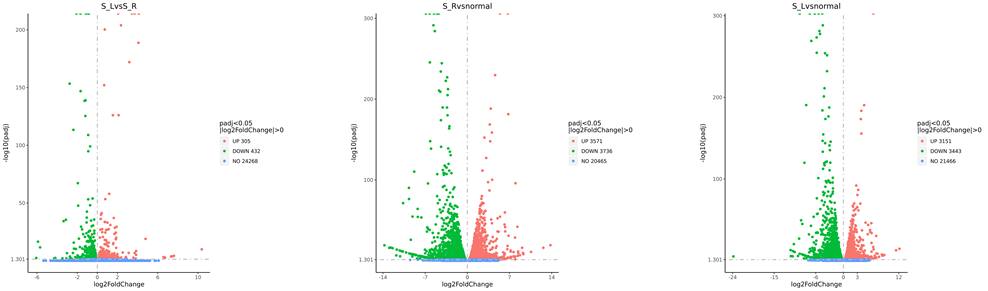


Supplemental Fig. 4. The gene expression of the lesions from both sides. The difference of gene expression between the lesions from both sides was not obvious, while the difference of gene expression between the lesions and healthy samples was obvious.


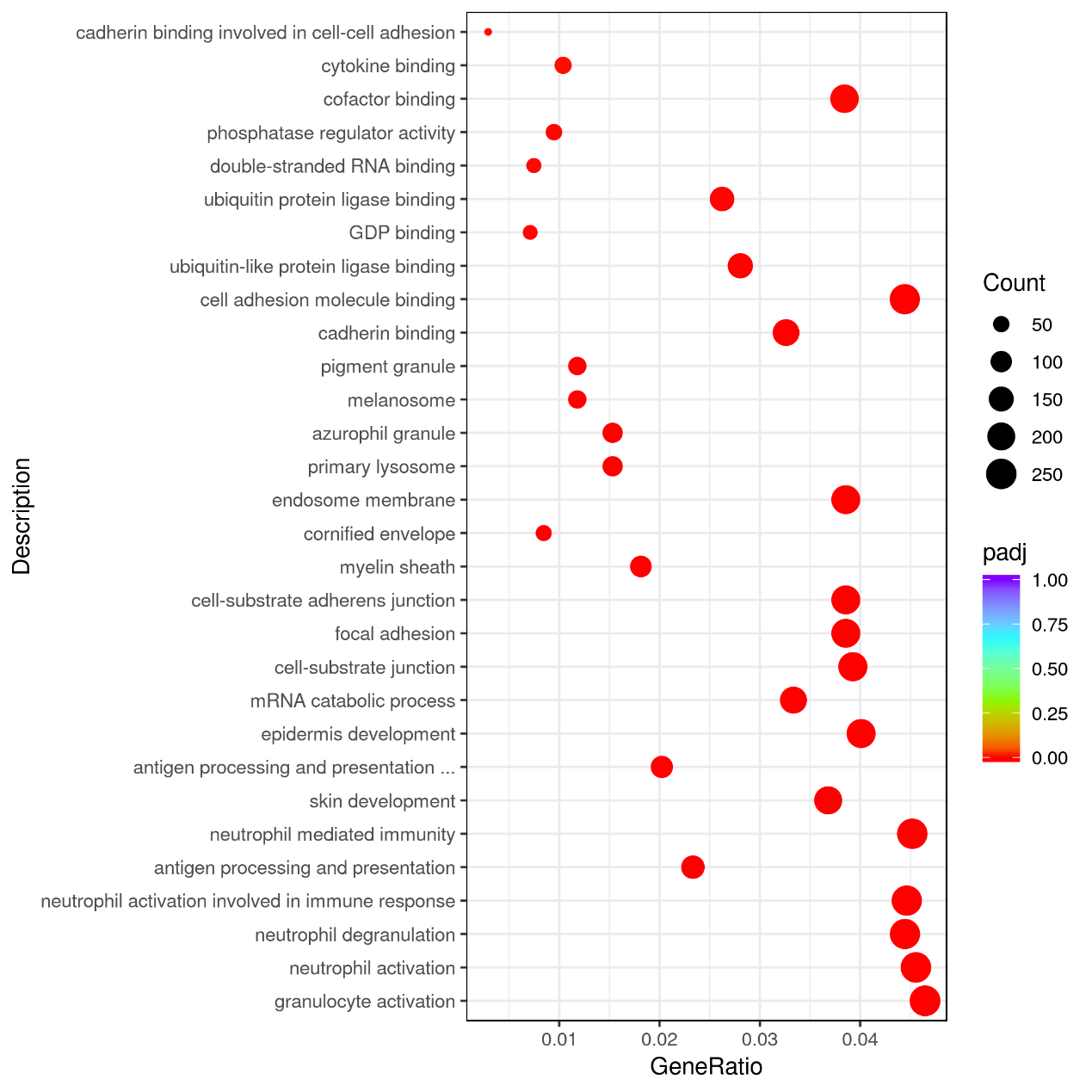


Supplemental Fig. 5 The enrichment analysis aimed at detecting the variation of cell signaling pathways and biological functions among different groups in the left mammary area.


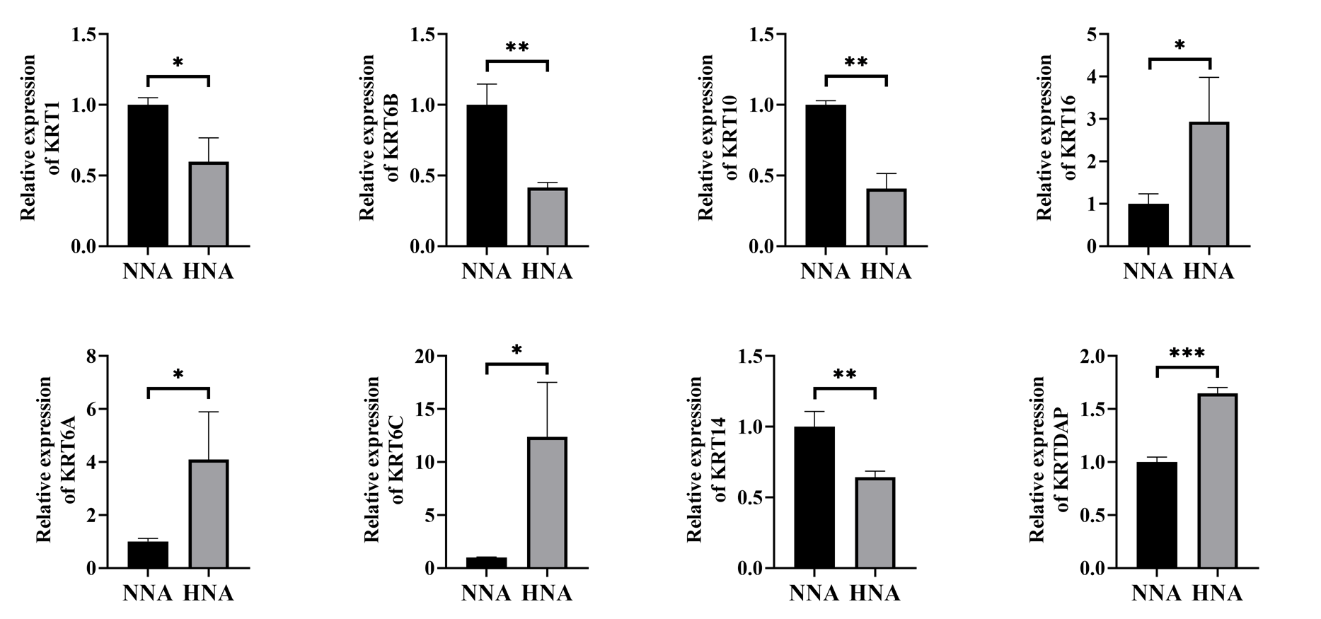


Supplemental Fig. 6. Keratins and their associated proteins dysregulated in HNA compared to NNA.
